# A new way of looking at transcription factor assays

**DOI:** 10.1038/s44320-024-00044-7

**Published:** 2024-06-07

**Authors:** Alan F Rubin

**Affiliations:** 1 Bioinformatics Division, Walter and Eliza Hall Institute of Medical Research, Parkville, Victoria Australia; 2 Department of Medical Biology, University of Melbourne, Parkville, Victoria Australia

**Keywords:** Chromatin, Transcription & Genomics

## Abstract

AF Rubin discusses a new high-throughput functional assay for transcription factors applied for a deep mutational scanning study of the transcription factor PAX6 by Kudla and colleagues (McDonnell et al, 2023) in this issue of *Molecular Systems Biology*.

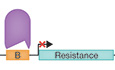

## Teaser

Advances in high-throughput functional genomics have enabled researchers to measure many thousands of individual genetic variants in a single gene in parallel using techniques such as deep mutational scanning (Fowler and Fields, 2014). The success of these approaches depends on the availability of assays that can measure a wide range of protein functions. In their recent work, Kudla and colleagues (McDonnell et al, 2024) applied deep mutational scanning to the transcription factor *PAX6*, which has a key role in eye development, and described a new high-throughput functional assay that could be applied to almost any transcription factor. The authors successfully measured the effects of 95% of missense variants in PAX6 and show that their assay results are highly concordant with known clinical variants. Notably, they also undertook a wide-ranging survey of computational variant effect predictors and show that their experimental data outperformed cutting-edge algorithms.

Deep mutational scanning and other multiplexed assays of variant effect have been applied to a wide variety of genes and other functional elements, and have been used for everything from biophysical studies to protein engineering to clinical variant classification. This family of methods share the same major steps: generation of a library of DNA variants, introduction of that library into an assay that enriches for functional variants, quantification of the resultant variant frequencies with high-throughput sequencing, and finally data analysis and interpretation (Tabet et al, 2022). Human or yeast cells are most often used for the assay system and the effect of each variant is commonly measured by observing its impact on cell growth or survival over time. These holistic phenotypes have the advantage of capturing multiple aspects of cell biology, but can make discerning the specific underlying mechanisms more challenging.

One powerful aspect of these approaches is the potential to combine high-throughput genomic methods with well-established, lower-throughput functional assays. Here, the researchers applied existing deep mutational scanning techniques with a yeast one-hybrid assay (Li and Herskowitz, 1993) to measure the ability of each variant to bind to PAX6 DNA response elements. To accomplish this, the DNA binding domain of PAX6 was expressed as a fusion protein with a yeast transcription activating domain. Variants that were competent binders drove expression of an antibiotic resistance gene, enabling cell growth, but cells containing a loss-of-function variant died. Moreover, this system allowed the identification of possible gain-of-function variants that had stronger expression than wild type. The authors assayed the same variant library against two different PAX6 response elements, one natural and one synthetic. This allowed them to differentiate between variants that affected DNA binding in general and variants that had a sequence-specific effect.

**Figure 1:**
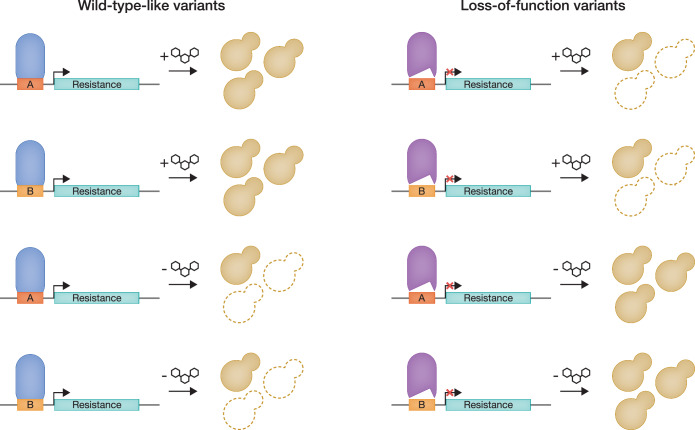
Cartoon representation of the four PAX6 deep mutational scanning assays. The left side shows the typical result for a wild-type-like variant that can bind DNA, and the right side shows the typical result for a loss-of-function variant. Each row shows one assay condition—one of two PAX6 binding elements in the presence or absence of antibiotics. Results were highly correlated for the two DNA binding elements, but anti-correlated for the different antibiotic treatments.

Surprisingly, when the experiment was performed in the absence of antibiotic, an inverted growth phenotype was observed (Fig. 1). PAX6 variants that were able to bind DNA had a detrimental effect on yeast growth, presumably due to promiscuous binding and general dysregulation of the yeast transcriptome. This suggests that other transcription factors could be assayed simply by measuring the growth defect without needing to first identify a suitable response element, a step that can present technical challenges. More work is needed to determine whether this is a general property of transcription factors in this experimental system or whether it is specific to *PAX6* or some subset of genes that bind DNA.

Transcription factors are clearly a large and important class of genes to understand (Lambert et al, 2018), and over 400 (including *PAX6*) have been identified as clinically relevant according to the widely-used Gene Curation Collection (GenCC) database of clinical genes (DiStefano et al, 2022). Because deep mutational scanning can experimentally measure all possible single amino acid or single nucleotide variants in a gene, it has become important to the clinical genomics community who need additional independent sources of data to help classify patient variants and inform diagnosis or treatment. The saturation nature of these experiments means that effects are measured even for very rare variants, allowing classification of a large proportion of variants observed in patients (Fayer et al, 2021).

Despite being collected in yeast, this PAX6 dataset achieved an accuracy of 92% for predicting whether previously-classified clinical variants were pathogenic or benign. The authors applied clinical best practice to formally evaluate the strength of clinical evidence for this assay (Brnich et al, 2019), annotating over 2000 PAX6 variants that can now be used by clinical variant curators. The researchers took the further step of investigating a previously-characterized cohort of patients with ocular disease and PAX6 missense variants. They found that patients with atypical phenotypes carried variants that produced more extreme results in their yeast one-hybrid assay.

The experimental assay also performed very well when compared to computational variant effect predictors. The team compared their deep mutational scanning results to the PAX6 predictions of over 50 algorithms, outperforming all unsupervised methods and only just falling short of the top supervised methods. This is especially impressive given that, as part of their training, supervised prediction methods may have already encountered the clinical variant data used for evaluation (Livesey and Marsh, 2023). Studies of these performance differences may help build improved variant effect prediction methods in the future.

As the field moves towards building a comprehensive atlas of human variation, we will need to continue to develop and deploy new methods that allow researchers to access different aspects of biology (Fowler et al, 2023). Expanding the repertoire of functional assays will help improve other applications of deep mutational scanning as well. Kudla and colleagues (McDonnell et al, 2024) provide a system that can efficiently evaluate thousands of transcription factor variants against multiple target sequences. If the results generalize to other genes, they could lead to new and exciting insights into transcription factor biology and patient diagnosis.
